# Synergistic enhancement of antioxidant and antiapoptotic levels by Vitamin A and Lupeol improves the quality of cryopreserved bucks semen

**DOI:** 10.3389/fvets.2026.1746386

**Published:** 2026-02-13

**Authors:** Congliang Wang, Xiayu Yun, Yanfeng Song, Minying Ju, Meixia Wang, Xiaoyue Song, Helin Li, Na Li, Lei Qu, Jinlian Hua, Xiaomin Du, Haijing Zhu

**Affiliations:** 1 Shaanxi Provincial Engineering and Technology Research Center of Cashmere Goats, College of Advanced Agricultural Sciences, Yulin University, Yulin, Shaanxi, China; 2 Shaanxi Centre of Stem Cells Engineering & Technology, Northwest Agriculture & Forestry University, Yangling, Shaanxi, China; 3 Yulin Animal Husbandry and Veterinary Service Center, Yulin Animal Husbandry and Veterinary Bureau, Yulin, Shaanxi, China; 4 College of Advanced Agricultural Sciences, Yulin University, Yulin, Shaanxi, China

**Keywords:** buck, cryopreservation, Lupeol, semen, Vitamin A

## Abstract

This study investigated the effects of supplementing bucks semen extenders with Vitamin A (Vit A) and Lupeol (Lup) on post-thawing quality. Pooled semen from six bucks was cryopreserved using extenders containing varying concentrations of Vit A and Lup. Post-thawing assessments included sperm kinematic parameters, plasma membrane and acrosome integrity, antioxidant enzyme activities, and the expression of antioxidant and apoptosis related genes. The results showed that the addition of 0.75 μM Vit A significantly improved post-thaw sperm motility, kinematic velocities (VSL, VCL, VAP), membrane integrity, and acrosome integrity compared to the control. Compared with the control group, this group also exhibited the significantly improved activities of SOD, CAT, and GSH-Px, along with significantly upregulated expression of the antioxidant genes *SOD1* and *NRF2*. Based on these findings, we further evaluated Lup. Compared with the control group, the addition of 2 μM Lup significantly enhanced sperm motility, VSL, WOB, and antioxidant enzyme activity. It also increased the expression of *SOD1*, *NRF2*, *GPX4*, and *CAT*, while reducing the transcriptional levels of the apoptotic genes *Caspase 3* and *P53*. Furthermore, compared to fresh semen, frozen semen supplemented with 0.75 μM Vit A and 2 μM Lup exhibited good fertilization capacity, although no significant differences were observed in cleavage rate or blastocyst rate. In conclusion, Vit A and Lup act synergistically in bucks semen cryopreservation. They enhance antioxidant capacity, suppress apoptosis-related gene expression, and mitigate oxidative stress during freeze-thawing, thereby improving semen quality.

## 1 Introduction

Semen cryopreservation plays a pivotal role in animal genetic improvement. It facilitates the cross-regional dissemination of high-quality germplasm resources through artificial insemination (AI) and intracytoplasmic sperm injection (ICSI). This technology overcomes geographical constraints in breeding applications. It also ensures the preservation of germplasm resources for endangered species and contributes to biodiversity conservation [1]. However, the cryopreservation process can induce ultrastructural and functional damage to spermatozoa. Specifically, cryostress increases plasma membrane permeability, which in turn promotes excessive generation of reactive oxygen species (ROS) and exacerbates lipid peroxidation (LPO). This oxidative assault also impairs mitochondrial function and damages polyunsaturated fatty acids within the sperm membrane. Consequently, acrosomal damage, DNA fragmentation, and altered membrane integrity occur, ultimately compromising semen quality and reproductive efficiency [2].

Compared with commercially available cryopreserved bovine and equine semen, caprine semen exhibits higher levels of cell membrane lipid content, which makes it more susceptible to cryodamage, including ice crystal formation, osmotic stress, and lipid peroxidation during the freezing process [3]. Furthermore, excessive amounts of phospholipase A in seminal plasma can combine with egg yolk to produce toxic substances such as lipid peroxides, which are detrimental to long-term semen preservation [4]. Furthermore, rapid temperature fluctuations during semen freezing and thawing can stimulate excessive production of ROS [5, 6]. After thawing, exposure to oxygen in the environment accelerates the generation of ROS, and antioxidant systems (such as the superoxide dismutase capacity in seminal plasma and sperm cells) cannot keep up with the rate of accumulation of hydroxyl and superoxide radicals [7]. Therefore, the addition of exogenous antioxidants to semen cryopreservation extenders can effectively enhance the antioxidant capacity of sperm cells, mitigate oxidative stress, and improve post-thaw sperm quality [8, 9]. To date, compounds such as glutathione [10], quercetin [11], and coenzyme Q10 [12] have been demonstrated to effectively reduce excessive reactive oxygen species accumulation, maintain plasma membrane integrity, and improve semen quality along with subsequent embryonic developmental potential. Consequently, the supplementation of antioxidants during buck semen cryopreservation represents an effective strategy for enhancing post-thawing semen quality.

Vitamin A (Vit A) is a fat soluble vitamin essential for maintaining normal physiological functions in animals. It participates in many physiological processes such as germ cell proliferation and differentiation, immune regulation, organ development, and embryonic development [13, 14], and it efficiently scavenges lipid peroxidation radicals, hydroxyl radicals, and various ROS, thereby mitigating cellular damage caused by free radicals [15]. *β*-carotene, a precursor to Vit A, increases the antioxidant capacity of the testicles and improves sperm concentration and motility by increasing the levels of the antioxidant glutathione and antioxidant enzymes such as catalase (CAT), superoxide dismutase (SOD), and glutathione peroxidase 1 (GPX1), and improves sperm concentration and motility [16]. Furthermore, dietary supplementation of Vit A deficient rams effectively restored normal acrosin and plasminogen activator activities in spermatozoa [17].

Lupeol (Lup) is a pentacyclic triterpenoid compound widely found in various fruits, vegetables, and medicinal plants. It effectively inhibits tumor cell proliferation, invasion, and metastasis, while also inhibiting tumor cell survival [18]. Additionally, it exhibits multiple biological functions, including immunomodulation and promotion of wound healing [19]. Exhibits important antioxidant and neuroprotective properties by inhibiting the generation of ROS and reducing oxidative burden in Alzheimer’s disease [20], and it attenuates ROS/lipid peroxidation (LPO) production induced by central nervous system injury, enhances antioxidant protein levels, and suppresses the expression of mitochondrial apoptotic signaling molecules such as Caspase 3 and Bax [21].

In summary, the antioxidant properties of Vit A and Lup, including their ability to scavenge ROS and mitigate oxidative stress, have been well documented, however, no studies have been reported on the simultaneous supplementation of these compounds in cryopreserved buck semen. This study was conducted to evaluate the effects of varying concentrations of Vit A and Lup on the preservation efficacy of cryopreserved buck semen, with the aim of providing scientific evidence to support the widespread application of frozen buck semen and enhance reproductive efficiency.

## 2 Materials and methods

### 2.1 Experimental animals

All experimental bucks were provided by the demonstration goat farm breeding base of Yulin University. Six healthy adult shaanbei white cashmere bucks, aged 2–4 years, were randomly selected for this study. All bucks had normal reproductive organs and were maintained under uniform feeding conditions, receiving the same diet and having free access to water.

The study was carried out from august to september, corresponding to the breeding season of shaanbei white cashmere goats. The experiment took place at the College of Advanced Agricultural Sciences of Yulin University, situated at an altitude of approximately 1,220 m, with coordinates of 38°43′N, 109°72′E. During this period, the average ambient temperature ranged from 22 °C ~ 27 °C, with an average relative humidity of 55% ~ 65%.

### 2.2 Experimental design

3.1 g glucose (Solarbio, #G8151, China), 4.6 g lactose (Solarbio, #L8911, China), 1.5 g sodium citrate (Solarbio, #S6100, China), 100,000 IU penicillin sodium (Yuanye, #B25911, China), and 100,000 IU streptomycin sulfate (Yuanye, #S17058, China) were accurately weighed and dissolved in 80 mL of double-distilled water using a constant temperature magnetic stirrer. The solution was then filtered through a 0.22 μm membrane filter into a sterilized glass bottle and adjusted to a final volume of 100 mL. After sterilization in a constant-temperature water bath at 60 °C for 30 min, the solution was cooled to room temperature. Subsequently, 15% egg yolk and 4% glycerol (Extender I) were added, and the final solution was stored at 4 °C.

Optimization of Vit A and Lup concentrations for cryopreservation of bucks semen. Vit A and Lup were purchased from Shanghai Yuanye Bio Technology Co., Ltd. (Shanghai, China). To determine the optimal concentration of Vit A in Extender I, a control group without Vit A supplementation was compared with groups supplemented with 0.25 μM, 0.50 μM, 0.75 μM, or 1.00 μM Vit A, five replicates were conducted. Using the optimal Vit A concentration as the base solution (Extender II), the effects of Lup on semen cryopreservation were investigated. The control group received no Lup, while the experimental groups were supplemented with 1 μM, 2 μM, and 4 μM Lup, five replicates were conducted. Finally, the fertilization capacity of oocytes and embryonic developmental competence were evaluated among fresh semen, cryopreserved semen with 0.75 μM Vit A, and cryopreserved semen with 2 μM Lup (the optimal concentration as determined experimentally).

### 2.3 Semen collection

Semen was collected from six bucks twice weekly for five weeks using an artificial vagina, one week prior to the formal experiment, semen collection was similarly performed to eliminate dead sperm. A total of 60 semen samples were obtained. The collected semen was transported to the laboratory within 30 min and evaluated for quality parameters such as sperm motility and density under a phase-contrast microscope. Samples meeting the following criteria were selected for subsequent experiments: semen volume of 0.8–1.5 mL, sperm motility ≥ 85%, and sperm concentration ≥ 2 × 10^9^ sperm cell/mL. To minimize individual variation, qualified semen from each collection was resuspended and homogenized in sterilized centrifuge tubes, and a total of 10 pooled semen samples were prepared.

### 2.4 Sperm cryopreservation and thawing

The collected semen samples were equally divided into five aliquots. Both the semen and extenders were pre-warmed at 37 °C. The semen was diluted 10-fold in 50 mL centrifuge tubes using an extender containing specified concentrations of Vit A or Lup. The diluted semen was loaded into 0.25 mL straws and equilibrated at 4 °C for 2 h. After equilibration, the straws were placed in a programmable freezer (Freeze Control, Cryologic, Australia) pre-cooled to 5 °C, the temperature was lowered from room temperature to −80 °C at a rate of 1–2 °C per min, after which the straws were plunged into liquid nitrogen for long-term storage. After three weeks of cryopreservation, the frozen straws were removed from the liquid nitrogen and thawed in a 37 °C water bath for 30 s, then incubated at 37 °C for subsequent use.

### 2.5 Semen quality analysis

Sperm motility parameters were assessed using a computer-assisted sperm analysis (CASA) system (CEROS II, Hamilton Thorne, Inc., Beverly, MA, USA). The system settings were configured as follows: phase-contrast imaging mode, frame rate of 60 Hz, number of frames 30 frames per analysis. Thawed semen was diluted with pre-warmed PBS to adjust the sperm concentration to 2–4 × 10⁶ sperm cell/mL. A 10 μL aliquot of the diluted sample was loaded into 37 °C pre-warmed Leja 4-chamber slide (Leja Products, Luzernestraat B. V., Holland) and gently covered with a coverslip (24 × 24 mm). Under a phase-contrast microscope (10 × objective), at least five fields were observed, with each field containing no fewer than 200 spermatozoa. The following kinematic parameters were assessed: Total motility (TM, %, percentage of spermatozoa with an average path velocity greater > 10 μm/s), straight line velocity (VSL, μm/s), curvilinear velocity (VCL, μm/s), average path velocity (VAP, μm/s), linearity (LIN, VSL/VCL, %), wobble (WOB, VAP/VCL, %).

### 2.6 Assessment of sperm plasma membrane integrity, and acrosome integrity

**Figure 1:**
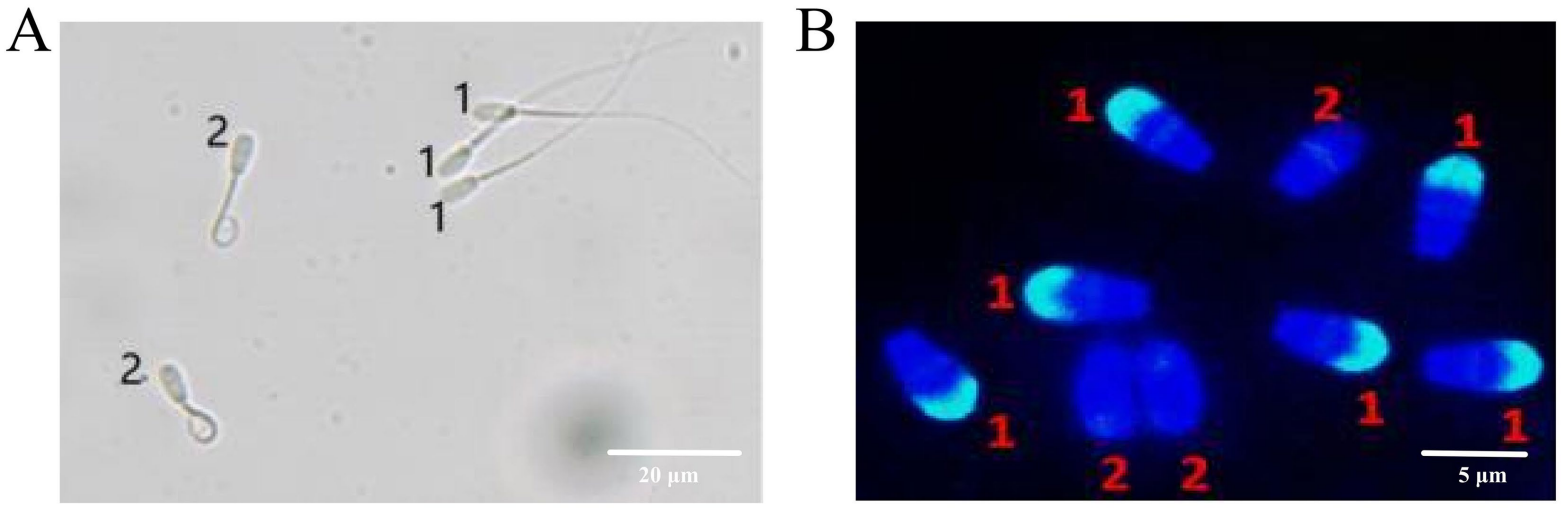
Assessment of sperm plasma membrane integrity and acrosome integrity. **(A)** Evaluation of sperm plasma membrane integrity by the hypoosmotic swelling test. Label 1 indicates a damaged plasma membrane, while label 2 denotes an intact plasma membrane. **(B)** Detection of sperm acrosome integrity using FITC-PNA staining. Label 1 represents an intact acrosome, whereas label 2 indicates a damaged acrosome.

Sperm plasma membrane integrity was assessed using the hypo-osmotic swelling test, with integrity determined according to the criteria illustrated in Figure 1A. A 900 μL aliquot of hypo-osmotic solution (prepared by dissolving 0.9 g fructose (Solarbio, #F8100, China) and 0.49 g sodium citrate (Solarbio, #S6100, China) in 100 mL deionized water) was pre-warmed at 37 °C for 30 min in a constant temperature water bath. Subsequently, 10 μL of the semen sample was pipetted onto a pre-warmed glass slide, gently covered with a coverslip, and the number of spermatozoa exhibiting coiled or swollen tails was counted under a microscope.

Sperm acrosome integrity was assessed using a fluorescein isothiocyanate peanut agglutinin (FITC-PNA) staining kit, with the evaluation criteria illustrated in Figure 1B. A 10 μL aliquot of the 10-fold diluted semen sample was smeared onto a glass slide and air-dried. The smear was fixed with pre-cooled methanol and dried. Then, 10 μL of FITC-PNA solution was applied, incubated at 37 °C in the dark for 30 min, and examined under an inverted fluorescence microscope. Spermatozoa displaying uniform and bright green fluorescence in the head region were classified as having intact acrosomes, while those without green fluorescence were considered acrosome-defective [10].

Sperm plasma membrane integrity and acrosome integrity were assessed by randomly selecting three microscopic fields, with each field containing at least 200 spermatozoa.

### 2.7 Determination of SOD, CAT and glutathione peroxidase (GSH-P_X_) concentration

After thawing, 200 μL of semen was transferred to a 1.5 mL centrifuge tube and centrifuged at 1600 × g for 10 min at 4 °C. The supernatant was discarded, and the sperm pellet was retained. Then, 200 μL of lysis buffer was added to lyse the pellet. After centrifugation at 12000 × g for 10 min at 4 °C, the resulting supernatant was collected for subsequent analysis. Assay kits for SOD, CAT, and GSH-PX were all purchased Beyotime Biotechnology Co., Ltd. (Shanghai, China). The detection results were calculated and recorded in strict accordance with the manufacturer instructions, as previously described [22, 23]. All measurements were performed in triplicate.

### 2.8 RNA extraction and quantitative real-time PCR (qRT-PCR)

Total RNA was extracted from sperm samples using TRIzol reagent (Takara, Dalian, China), which contains phenol, guanidine isothiocyanate (GITC), and dithiothreitol (DTT), according to the manufacturer protocol. Briefly, 1 mL of preheated 65 °C TRIzol was added to the sperm pellet, thoroughly mixed, and vortexed for 3 min. Then, 20 μL of *β*-mercaptoethanol was added, and the mixture was incubated in a 65 °C water bath for 30 min. Afterward, 200 μL of chloroform was added, and the mixture was incubated on ice for 15 min, followed by centrifugation at 20,000 rpm for 15 min at 4 °C. The aqueous phase was carefully transferred to a new RNase-free tube, and an equal volume of isopropanol was added for RNA precipitation overnight at −20 °C. The next day, the sample was centrifuged at 20,000 rpm for 15 min at 4 °C. The supernatant was discarded, and the RNA pellet was washed twice with 75% ethanol at 4 °C and finally resuspended in RNase-free water.

RNA integrity was evaluated by electrophoresis on a 1.5% non-denaturing agarose gel. Clear 28S and 18S rRNA bands were observed, with the 28S band approximately twice as intense as the 18S band, confirming high-quality RNA. First-strand cDNA synthesis was performed using the HiScript III 1st Strand cDNA Synthesis Kit (Vazyme, Nanjing, China). For each reaction, 2 μg of total RNA was incubated with 4 μL of 5 × gDNA wiper Mix at 42 °C for 3 min to remove genomic DNA. Then, 4 μL of HiScript III Enzyme Mix was added to the same tube, and reverse transcription was carried out at 50 °C for 15 min, followed by enzyme inactivation at 85 °C for 5 s. The resulting cDNA was stored at −80 °C.

The expression of antioxidant and apoptosis-related genes in spermatozoa was analyzed, including superoxide dismutase 1 (*SOD1*), nuclear factor erythroid 2 related factor 2 (*NRF2*), glutathione peroxidase 4 (*GPX4*), catalase (*CAT*), cysteine dependent aspartate specific protease 3 (*Caspase 3*), and protein p53 (*P53*). The qRT-PCR primers were designed using Primer Premier 5.0 software and synthesized by Sangon biotech Co., Ltd.(Shanghai, China). The primer sequences are listed in Table 1, qRT-PCR procedure and reaction system refer to the previous report [24]. A three-step amplification protocol protocol was employed, with glyceraldehyde-3-phosphate dehydrogenase‌ (*GAPDH)* as the internal control gene. The relative expression levels of target genes were determined using the 2 − ^ΔΔ^Ct method. All sample measurements were performed in triplicate.

**Table 1:** Primer sequence information.

Target genes	Primer sequence (5′ -3′)	Product length (bp)	Annealing temperature (°C)
GAPDH	F: AAAGTGGACATCGTTGCCAT R: CCGTTCTCTGCCTTGACTGT	116	60
SOD1	F: ATCCACTTCGAGGCAAAGGG R: TTCACATTGCCCAGGTCTCC	206	60
NRF2	F: AGTCCCGGTCATCGGAAAAC R: AGCTTTTGCCCGTAGCTCAT	93	60
GPX4	F: CAATGTGGCCTCGCAATGAG R: GCCAGGATCCGTAAACCACA	96	60
CAT	F: CACTCAGGTGCGGGATTTCT R: CTGGATGCGGGAGCCATATT	117	60
Caspase3	F: CAGACGTGGATGCAGCAAAC R: CATGGCTTAGAAGCACGCAA	165	60
P53	F: CAGCTCCTCTCCACAGCAAA R: AGAATGAGCCCTGCTTTCCC	163	60

### 2.9 Evaluation of IVF capacity

Ovaries were collected from adult does at a local slaughterhouse and transported to the laboratory in sterile physiological saline containing penicillin and streptomycin at 37 °C. The ovaries were then placed in a 60 mm cell culture dish. Within a laminar flow hood, follicles measuring 2–6 mm in diameter were dissected using a surgical scalpel. Cumulus-oocyte complexes (COCs) displaying two or more layers of compact cumulus cells and homogeneous, dense cytoplasm were selected under a stereomicroscope. The COCs were transferred to BO-IVM medium (IVF Bioscience, Falmouth, UK) under mineral oil at a density of 20–25 COCs per 100 μL. The cultures were incubated in a humidified atmosphere of 5% CO₂ at 38.5 °C for 22–25 h.

Sperm samples were centrifuged through a PureSperm™ 80/40 (Nidacon, Gothenburg, Sweden) gradient at at 1,500 rpm for 8 min. The resulting pellet was resuspended in pre-warmed BO-IVF medium (IVF Bioscience, Falmouth, UK) to a final concentration of 2 × 10^8^ sperm cell/mL. The COCs were transferred into BO-IVF medium under mineral oil at a density of 20–25 COCs per 100 μL. Then, 10 μL of the prepared sperm suspension was introduced into the BO-IVF medium, and fertilization was carried out in a humidified incubator at 38.5 °C with 5% CO₂ for 20–22 h. The cumulus cells were removed, and the fertilized oocytes were transferred into IVC medium (IVF Bioscience, Falmouth, UK). The day of fertilization was designated as Day 0, cleavage rates were assessed on Day 2, and blastocyst formation was recorded on Days 6 and 7. Each IVF experiment was repeated three times, with 45 oocytes used per replicate.

### 2.10 Data statistics and analysis

Statistical analyses were performed using IBM SPSS Statistics 26.0 (SPSS Inc., Chicago, IL, USA). The normality of the data was assessed with the Shapiro–Wilk test, and the homogeneity of variances was evaluated using Levene’s test. A one-way analysis of variance (ANOVA) was then applied, followed by post-hoc comparisons with the Least Significant Difference (LSD) test. The LSD test is a multiple comparison procedure that does not apply a strict correction to the significance level. Data visualization and histogram generation were performed using GraphPad Prism 8.0.1 software. Results are presented as mean values ± SEM, and *p* < 0.05 was considered statistically significant, while *p* < 0.01 was considered highly statistically significant. The *p* values reported are nominal, derived from the LSD post-hoc test.

## 3 Results

### 3.1 Effects of Vit A supplementation on post-thawing buck sperm quality

**Figure 2:**
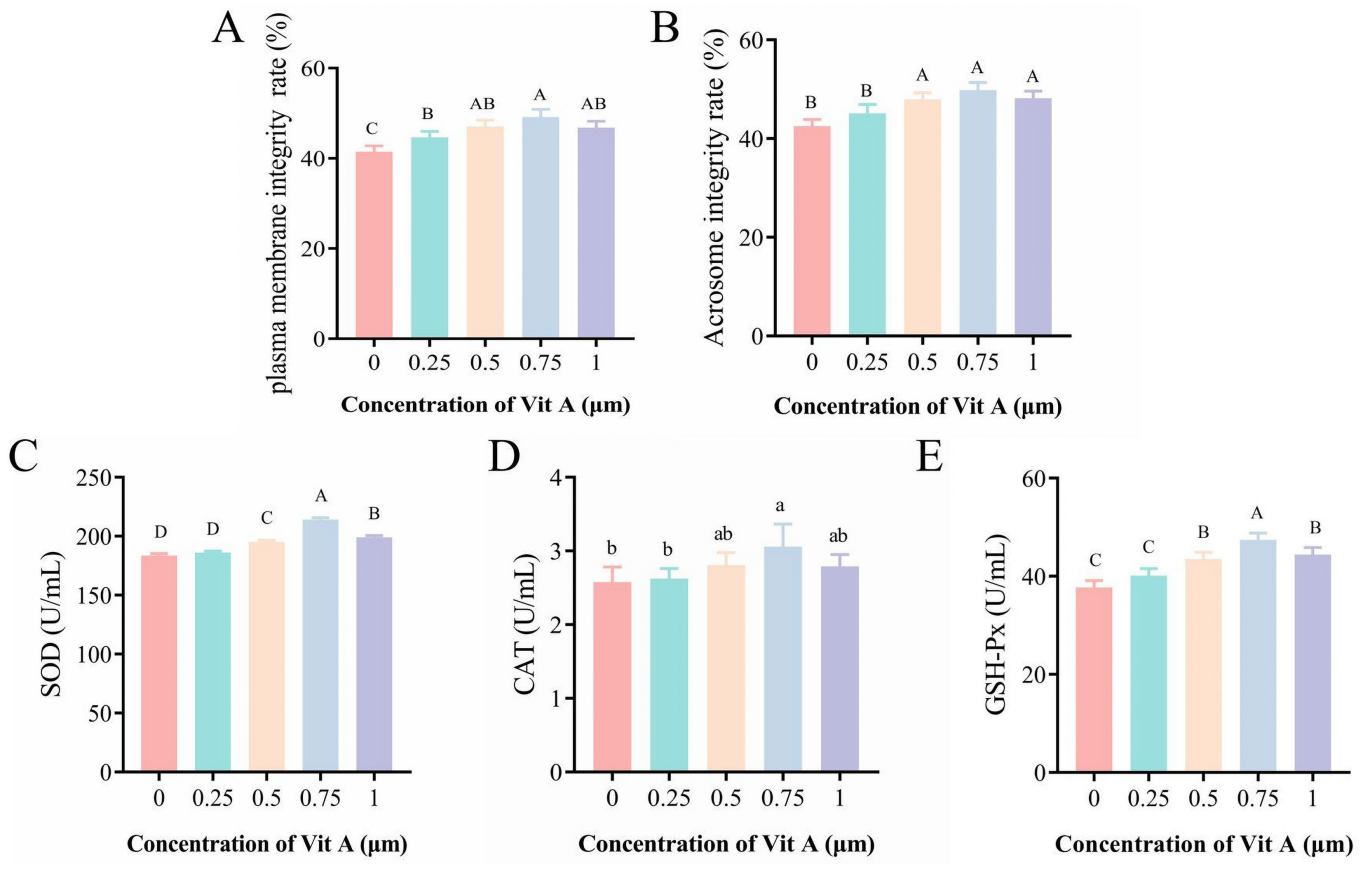
Effects of Vit A supplementation on sperm plasma membrane integrity rate, acrosome integrity rate, and antioxidant capacity after freeze-thawing. **(A,B)** Plasma membrane integrity and acrosome integrity after post-thawing. **(C–E)** Activities of SOD, CAT, and GSH-Px in sperm after post-thawing. Different lowercase letters indicate significant differences (*p* < 0.05), while different uppercase letters denote highly significant differences (*p* < 0.01). Mean values ± SEM are shown.

The effects of various Vit A concentration on post-thaw sperm motility and kinematic parameters are presented in Table 2. Sperm motility was significantly higher (*p* < 0.01) in all Vit A supplemented groups than in the control, and the VSL, VCL, and VAP values in the 0.75 μM Vit A group were also significantly elevated compared to the control and other groups (*p* < 0.01). However, no significant improvement was observed in the values of LIN and WOB (*p* > 0.05). Compared to the control group, 0.75 μM Vit A supplementation also significantly improved plasma membrane and acrosome integrity (*p* < 0.01; Figures 2A, 2B). Compared to the control and other group, the 0.75 μM Vit A group exhibited significantly higher higher SOD and GSH-Px activities (*p* < 0.01; Figures 2C, 2E), while CAT activity was also significantly higher than in the control (*p* < 0.05; Figure 2D).

**Table 2:** The effects of different concentrations of Vit A and Lup on the motility parameters of bucks semen.

Group	Parament					
	TM (%)	VSL (μm/s)	VCL (μm/s)	VAP (μm/s)	LIN (%)	WOB (%)
Vit A (μM)						
0	43.86 ± 0.49^D^	12.03 ± 0.99^C^	33.37 ± 2.91^B^	15.26 ± 0.31^B^	17.47 ± 2.49	23.38 ± 1.45
0.25	49.11 ± 0.38^C^	12.62 ± 0.30^BC^	35.69 ± 1.18^B^	14.97 ± 0.45^B^	17.04 ± 0.31	21.88 ± 0.76
0.5	52.59 ± 0.73^B^	13.73 ± 0.41^BC^	36.51 ± 1.55^B^	16.03 ± 0.95^B^	18.51 ± 0.25	23.46 ± 0.88
0.75	57.55 ± 0.77^A^	18.71 ± 0.73^A^	43.15 ± 0.99^A^	19.04 ± 0.38^A^	21.24 ± 1.37	23.82 ± 1.30
1	55.42 ± 1.31^A^	14.72 ± 1.08^B^	35.33 ± 1.67^B^	16.42 ± 0.95^B^	20.19 ± 1.57	23.81 ± 0.73
Lup + Vit A (μM)						
0	57.06 ± 0.68^BC^	14.83 ± 1.21^b^	38.36 ± 1.61	17.48 ± 1.38^ab^	18.39 ± 0.96^ab^	22.42 ± 1.09^b^
1	58.95 ± 0.42^B^	15.22 ± 1.33^ab^	42.61 ± 1.01	18.24 ± 0.93^ab^	17.33 ± 0.82^b^	22.93 ± 0.81^ab^
2	63.10 ± 1.08^A^	18.99 ± 1.42^a^	46.18 ± 3.55	20.55 ± 0.94^a^	21.47 ± 1.25^a^	25.76 ± 0.96^a^
4	55.70 ± 0.07^C^	13.13 ± 0.98^b^	35.88 ± 1.12	16.50 ± 0.52^b^	17.99 ± 1.30^ab^	24.16 ± 1.16^ab^

Each concentration of Lup contains 0.75 μM of Vit A. Mean values ± SEM are shown; Cells with different letters means *p* < 0.05 (a-b) and means *p* < 0.01 (A-D). TM, total motility; VSL, straight line velocity; VCL, curvilinear velocity; VAP, average path velocity; LIN, linearity, WOB, wobble.

### 3.2 Effects of Lup + Vit A supplementation on post-thawing buck sperm quality

**Figure 3:**
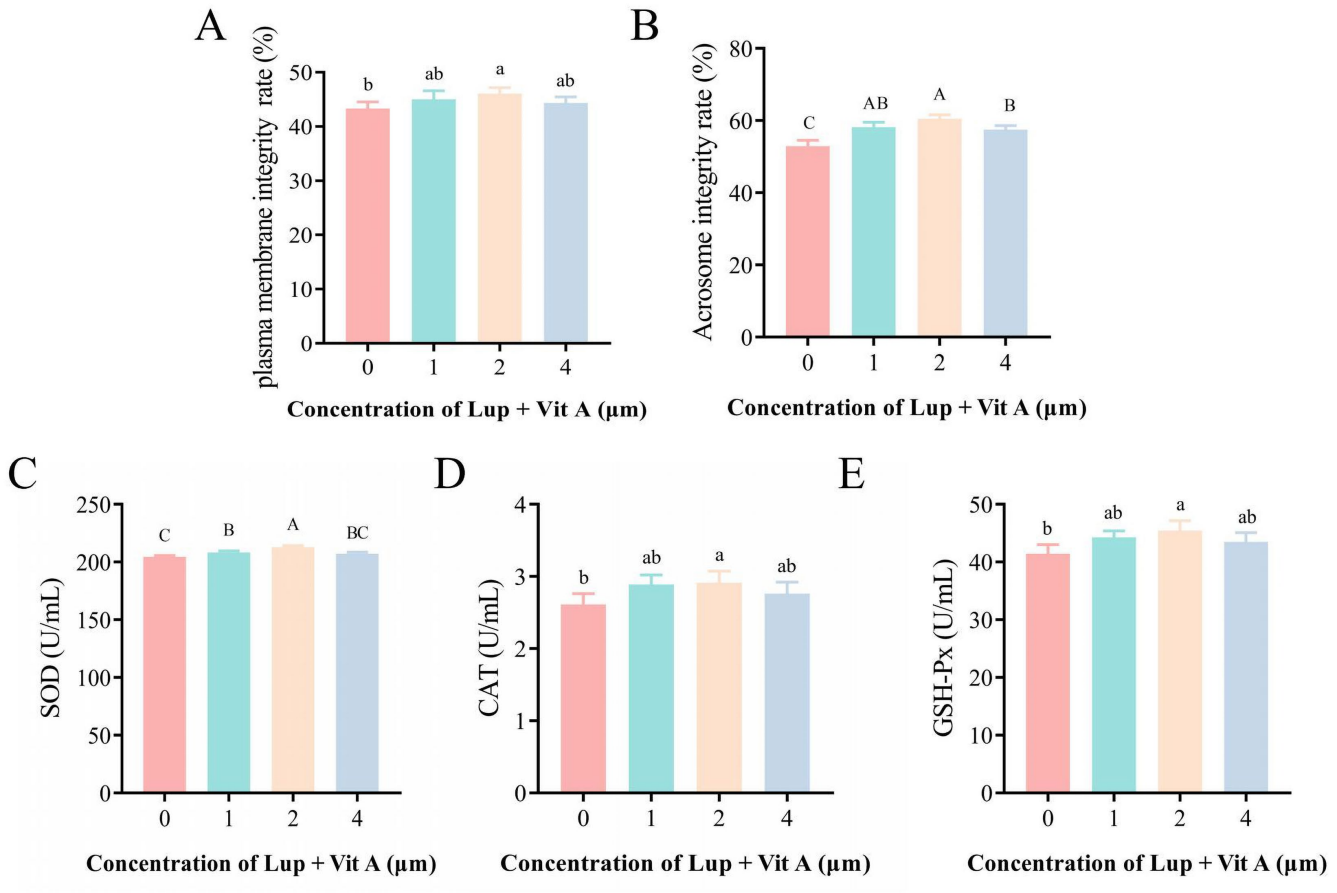
Effects of Lup + Vit A supplementation on sperm plasma membrane integrity rate, acrosome integrity rate, and antioxidant capacity after freeze-thawing. **(A,B)** Sperm plasma membrane integrity and acrosome integrity after post-thawing. **(C–E)** Activities of SOD, CAT, and GSH-Px in sperm after post-thawing. Different lowercase letters indicate significant differences (*p* < 0.05), while different uppercase letters denote highly significant differences (*p* < 0.01). Note: Each concentration of Lup contains 0.75 μM of Vit A. Mean values ± SEM are shown.

The effects of different concentrations of Lup on post-thaw sperm motility and kinematic parameters are presented in Table 2. The group supplementation with 2 μM Lup + 0.75 μM Vit A showed a highly significant improvement in sperm motility compared to the control and other groups (*p* < 0.01). This group also exhibited significantly increased VSL and WOB values compared to the control (*p* < 0.05). Compared with the control, the 2 μM Lup + 0.75 μM Vit A group demonstrated significantly increased plasma membrane integrity (*p* < 0.05; Figure 3A) and highly significantly improved acrosome integrity (*p* < 0.01; Figure 3B). Furthermore, compared to the control and other group, the 2 μM Lup + 0.75 μM Vit A group showed a highly significant increase in superoxide dismutase (SOD) activity (*p* < 0.01; Figure 3C), along with significant higher than the control in catalase (CAT) and glutathione peroxidase (GSH-Px) activities (*p* < 0.05; Figures 3D, 3E).

### 3.3 Effects of Vit A and Lup + Vit A supplementation on antioxidant capacity and apoptosis related gene expression in post-thawing buck sperm

**Figure 4:**
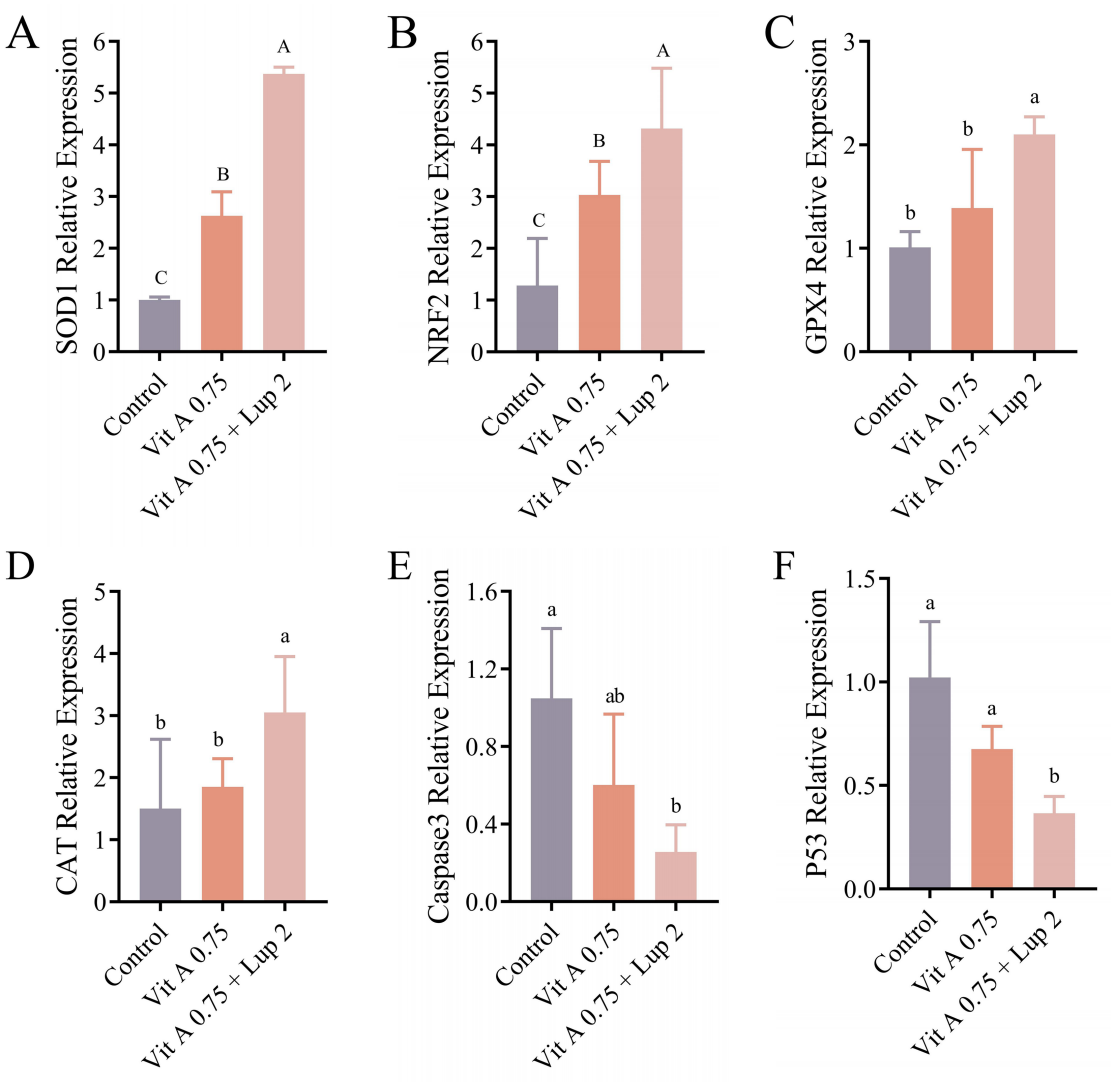
Effects of Vit A and Lup + Vit A supplementation on the mRNA expression of antioxidant and apoptosis related genes in sperm after freeze-thawing. **(A–D)** mRNA expression levels of *SOD1*, *NRF2*, *GPX4*, and *CAT* in post-thawing spermatozoa. **(E,F)** mRNA expression levels of *Caspase 3* and *P53* in post-thawing spermatozoa. Different lowercase letters indicate significant differences (*p* < 0.05), while different uppercase letters denote highly significant differences (*p* < 0.01). Note: Each concentration of Lup contains 0.75 M of Vit A. Mean values ± SEM are shown.

The effects of different concentrations of Vit A and Lup + Vit A on antioxidant capacity and apoptosis-related gene expression in post-thaw bucks sperm are showed in Figure. 4. The mRNA expression levels of antioxidant genes *SOD1* and *NRF2* were significantly upregulated in the 0.75 μM Vit A and 2 μM Lup + 0.75 μM Vit A groups compared to the control group (*p* < 0.01; Figures 4A, 4B). The 2 μM Lup + 0.75 μM Vit A group also showed significantly increased expression of *GPX4* and *CAT* (*p* < 0.01, Figures 4C, 4D), whereas the expression of apoptosis-related genes *Caspase 3* and *P53* was significantly downregulated (*p* < 0.05, Figures 4E, 4F).

### 3.4 *In vitro* fertilization and embryonic developmental competence of cryopreserved semen

**Figure 5:**
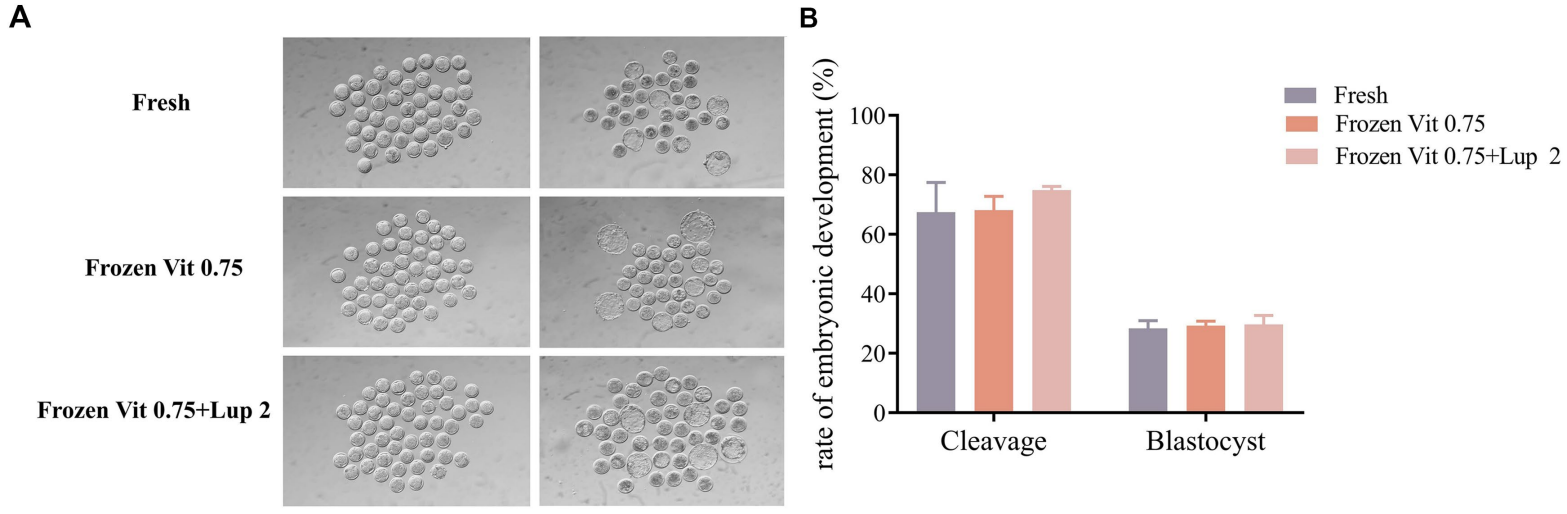
Comparison of *in vivo* fertilization efficiency between fresh semen and frozen-thawing semen. **(A)** Cleavage and blastocyst development rates of IVF embryos. **(B)** Embryonic developmental rates. Note: Each concentration of LUP contains 0.75 μM of Vit A. Mean values ± SEM are shown.

Mature oocytes were fertilized *in vitro* using fresh semen, semen with 0.75 μM Vit A, and semen with 2 μM Lup + 0.75 μM Vit A, and the embryonic cleavage and blastocyst formation rates were evaluated across the groups. As shown in Figure 5, neither cleavage nor blastocyst formation rates differed significantly (*p* > 0.05) among the fresh semen, 0.75 μM Vit A, and 2 μM Lup + 0.75 μM Vit A groups.

## 4 Discussion

The widespread application of semen cryopreservation technology has effectively prolonged sperm lifespan, overcoming temporal and geographical constraints while maximizing the preservation of superior genetic resources from elite breeding males. Although significant progress has been made in buck semen cryopreservation techniques, critical challenges such as reduced post-thawing sperm motility, impaired sperm function, and oxidative stress remain unresolved [25]. Consequently, the supplementation of exogenous antioxidants is a pivotal strategy for enhancing the quality of cryopreserved semen and fertilization efficiency [26]. In this study, we investigated the effects of varying concentrations of Vit A and Lup added to the cryopreservation extender on post-thawing buck semen quality, and evaluations included sperm kinematic parameters, antioxidant enzyme levels, and mRNA expression of antioxidant and apoptosis related genes. In addition to comparisons with published literature [23, 27], we further conducted in vitro fertilization assays using fresh semen, Vit A supplemented cryopreserved semen, and cryopreserved semen cosupplemented with Vit A and Lup to validate sperm fertilization efficiency and subsequent embryonic developmental competence.

During artificial insemination in does, sperm must traverse the folded cervical os and the narrow, elongated oviduct, therefore, sperm TM is the primary indicator for evaluating semen quality [28]. Studies have shown that VCL, VAP, VSL, and LIN in the CASA system of bucks sperm are significantly positively correlated with migration efficiency in cervical mucus, while in ram sperm, only VCL and VAP exhibit this relationship. However, parameters reflecting more detailed kinematic characteristics, such as ALH, did not correlate with migration efficiency in either species [29]. These results identify key CASA parameters, VSL, VCL, VAP, and LIN, as core indicators for assessing the fertilization potential of buck semen. This aligns with our central objective of enhancing fertilization efficiency for practical application. Thus, we focused our analysis on TM, VSL, VCL, VAP, and LIN. In our study, supplementation with 0.75 μM Vit A significantly improved sperm motility compared to the control group. Specifically, the parameters VSL, VCL, and VAP increased markedly. This treatment also significantly enhanced sperm plasma membrane integrity, acrosome integrity, and overall antioxidant capacity. Concurrently, the levels of key antioxidant enzymes (SOD, CAT, and GSH-Px) were significantly elevated. As a fat soluble vitamin, Vit A readily penetrates the cell membrane and scavenges lipid peroxidation derived free radicals and other reactive species by regulating the activation of stimulated by retinoic acid 6 (STRA6), thereby alleviating oxidative stress [30]. Concurrently, it activates the p38 MAPK-PGC-1α pathway, resulting in enhanced mitochondrial membrane potential, suppression of the mitochondrial permeability transition pore opening, diminished mitophagy, and higher mitochondrial DNA copy number [31], and these mechanisms collectively restore mitochondrial function and maintain energy homeostasis, ensuring a sustained supply of ATP required for sperm motility. Additionally, Vit A significantly reduces intracellular lipid droplet accumulation by activating transcription factor SKN-1, supports mitochondrial membrane integrity, and enhances resistance to oxidative stress, thereby protecting the sperm membrane from peroxidative damage and maintaining intracellular homeostasis [32]. Based on the aforementioned findings, we speculation that this mechanism may be crucial in explaining how Vit A supplementation enhances the quality of cryopreserved bucks semen.

Although sperm motility did not differ significantly between the 0.75 μM and 1 μM Vit A treatments, the 0.75 μM group exhibited significantly higher VSL, VCL, VAP, SOD, and GSH-Px enzyme activities. Consequently, the 0.75 μM Vit A concentration was selected to investigate whether a combined supplement of Vit A and Lup could synergistically enhance bucks semen cryopreservation. The results demonstrated that the addition of 0.75 μM Vit A + 2 μM Lup to the semen cryopreservation extender significantly improved post-thawing sperm motility parameters, plasma membrane integrity, and acrosome integrity. In fresh semen, SOD converts superoxide anions into hydrogen peroxide, CAT further decomposes hydrogen peroxide, and GSH-Px utilizes glutathione as a substrate to reduce hydrogen peroxide or organic peroxides [33]. These antioxidant enzymes work synergistically to protect sperm membrane integrity and maintain intracellular homeostasis. Our findings demonstrate that the combined supplementation of 2 μM Lup + 0.75 μM Vit A significantly increased the activity of key of antioxidant enzymes, including SOD, CAT, and GSH-Px, leading to enhancing free radical scavenging and inhibiting lipid peroxidation [34]. Eleraky et al. [35] reported that the active metabolite of Vit A, retinoic acid, can bind to nuclear receptors RAR/RXR to form a heterodimer, and this complex interacts with retinoic acid response elements (RAREs) in the promoter regions of antioxidant enzyme genes such as SOD and CAT, thereby activating their transcription. Meanwhile, Arumuganainar et al. [36] found that terpenoid like compounds such as Lup activate the Keap1-Nrf2-ARE pathway. This promotes nuclear translocation of Nrf2 and enhancing its binding to antioxidant response elements (AREs). Furthermore, these compounds stabilize enzyme protein conformations, upregulate the transcription and expression of SOD, CAT, and GSH-Px genes, and enhance enzymatic activity to eliminate ROS. Therefore, we speculate that Vit A and Lup act as activators that upregulate antioxidant enzyme activity, which may be a key reason for their synergistic enhancement of endogenous antioxidant defenses in sperm.

The transcriptional levels of antioxidant and apoptosis related genes in cryopreserved buck semen were examined. It was found that the combined supplementation of 2 μM Lup + 0.75 μM Vit A in semen cryopreservation extenders significantly upregulated the transcriptional levels of antioxidant genes such as *SOD1*, *NRF2* and *GPX4*, concurrently, a notable upregulated was also observed with Vit A supplementation alone, and these findings further confirm that the addition of Vit A and Lup enhances antioxidant enzyme levels. It has been established that Vit A can boost antioxidant defenses by stabilizing NRF2 and promoting its nuclear translocation [37]. Similarly, Lup exerts its antioxidative and cytoprotective effects via the upregulation of NRF2 [38]. As described earlier, ROS induce mitochondrial membrane damage, oxidative DNA base modifications, and double-strand breaks. These insults subsequently activate the P53 mediated intrinsic apoptotic pathway, culminating in increased sperm apoptosis [39, 40]. Our findings demonstrate that the combined supplementation of 2 μM Lup + 0.75 μM Vit A significantly downregulates the expression levels of *Caspase 3* and *P53* genes and inhibits sperm apoptosis compared to the control group. These results are consistent with the studies by Zhu et al. [41], which reported that exogenous antioxidant supplementation could improve frozen semen quality by upregulating antioxidant gene and downregulating apoptosis related gene expression. It is important to recognize that this study focused on transcriptional changes in antioxidant and apoptosis-related genes regulated by Vit A and Lup. However, mRNA levels do not invariably correlate with the activity of the corresponding proteins. To directly validate the proposed roles of Vit A and Lup in sperm cryopreservation, future work could employ western blot analysis to quantify the expression and activation states of key proteins, such as NRF2 and Caspase 3, along with their downstream effectors, in sperm following freeze–thaw. This would provide direct functional evidence to corroborate the present findings. Furthermore, while we evaluated the transcription of apoptosis-related genes in the frozen semen, we did not assess functional apoptotic indicators such as sperm DNA fragmentation or mitochondrial membrane potential. Inclusion of these parameters in future studies would allow for a more comprehensive assessment of the protective effects of Vit A and Lup against cryopreservation-induced apoptotic damage.

It has been reported that the levels of apoptosis markers exhibit a significant negative correlation with sperm capacity for oocyte penetration, Elevated concentrations of apoptotic markers are consistently associated with with impaired sperm-oocyte binding [42]. Furthermore, the activation of apoptosis markers such as *Caspase 3* and pronounced DNA fragmentation not only impair sperm motility but also reduce the success rates of sperm oocyte binding and fertilization, these pathological alterations consequently lead to increased incidence of embryonic mortality or malformation [43]. Studies confirm that conventional frozen semen prepared without cryoprotectants induces severe functional decline in spermatozoa due to oxidative damage and cold shock, markedly reducing both cleavage and blastocyst rates [44, 45]. Fresh semen, used here as a high-standard control with intact sperm function, directly reflects the overall impact of freezing protocols and additives on fertilization potential. We therefore performed *in vitro* fertilization of mature does oocytes using fresh semen, semen supplemented with 0.75 μM Vit A, and semen supplemented with 2 μM Lup + 0.75 μM Vit A. This was done to assess whether the optimized cryopreservation protocol yields embryo development outcomes comparable to those achieved with fresh semen following in vitro fertilization. The results showed that the cleavage and blastocyst development rates were similar for embryos derived from fresh semen and semen supplemented with vitamin A, either alone or in combination with Lup, but no significant. These results are consistent with previous research findings in dairy bucks [46]. This phenomenon may be attributed to two primary factors: first, the high quality sperm obtained through PureSperm™ density gradient centrifugation may play a crucial role, which has been demonstrated to significantly reduce apoptotic sperm and cellular debris while maintaining a viable sperm recovery rate exceeding 85% [47], second, the supplementation of Vit A and Lup may effectively enhance sperm motility, plasma membrane integrity, acrosome integrity, and antioxidant capacity while reducing sperm apoptosis levels.

This study evaluated the effects of Vit A and Lup on the cryopreservation efficacy of buck semen. The statistical analysis was based on n = 5 independent experimental replicates, which were generated by pooling semen samples from six bucks. This design minimized inter-individual variability and thereby enhanced the accuracy and consistency of detecting the effects of Vit A and Lup supplementation at the population level. Despite the moderate sample size, the pooling strategy effectively reduced within-group variation, revealing statistically significant differences in multiple key quality parameters such as sperm motility and antioxidant enzyme activities [46]. These results indicate that the study design provided sufficient statistical power to reliably reveal the main positive effects of the additives. Consequently, the study conclusions demonstrate good reproducibility under comparable experimental conditions. However, while the pooled sample design effectively assessed overall population level effects, it also masked potential individual variations in responses to the exogenous additives among different bucks. It should be noted that the statistical approach in this study did not include an adjustment for multiple comparisons. It is acknowledged that this may elevate the Type I error rate, although the tests were confined to planned comparisons between specific groups. The application of more conservative methods (e.g., Bonferroni correction) in future research would strengthen the conclusions drawn from such findings. A another notable limitation of this study is the relatively small sample size, as semen samples were pooled from only six bucks. Future studies should therefore validate these findings using a larger cohort of individual animals. This would help to clarify the effects of the additives and improve the generalizability of the results across goats with diverse genetic backgrounds.

## 5 Conclusion

In summary, the addition of 0.75 μM Vit A and 2 μM Lup to bucks semen cryopreservation extenders significantly improved post-thawing sperm motility parameters, plasma membrane integrity, and antioxidant capacity, while reducing the proportion of apoptotic spermatozoa and may improve fertilization and early embryonic development *in vitro*, although further studies are needed to confirm this effect. Therefore, the supplementation of Vit A and Lup in semen cryopreservation extenders is beneficial for bucks breeding programs, providing a valuable reference for the commercial application of frozen semen and the preservation of elite germplasm resources in bucks.

## Data Availability

The original contributions presented in the study are included in the article/supplementary material, further inquiries can be directed to the corresponding author/s.
